# Enhancement of Antioxidant and Anti-Inflammatory Activities of Radish (*Raphanus sativus* L.) By-Products Through Enzymatic Pretreatment and Lactic Acid Fermentation

**DOI:** 10.3390/foods15071150

**Published:** 2026-03-27

**Authors:** Mi Hye Park, Kwang-Ok Kim

**Affiliations:** 1 Department of Food Science and Nutrition, Kyungpook National University, 80 Daehak-ro, Buk-gu, Daegu 41566, Republic of Korea; pmh704@gmail.com; 2 Department of Food and Nutrition, Gimcheon University, Gimcheon 39528, Republic of Korea

**Keywords:** radish by-products, enzymatic pretreatment, lactic acid fermentation, antioxidant activity, anti-inflammatory activity

## Abstract

Radish (*Raphanus sativus* L.) is an important vegetable resource in the food industry, generating substantial amounts of by-products during cultivation and distribution. Despite their richness in functional components, these by-products are largely underutilized. Accordingly, there is increasing interest in their valorization as functional food ingredients. This study evaluated the functional potential of radish by-products removed prior to distribution by applying cellulase pretreatment and *Lactiplantibacillus plantarum* fermentation individually or in combination. Radish samples were separated into leaf blade, stem, and taproot tissues and processed as untreated control, enzyme-treated, fermented, and enzyme-treated and fermented (EF) groups. The EF treatment significantly increased reducing sugar content, total polyphenols, and total flavonoids across all tissues, with the most pronounced enhancement observed in leaf by-products. In antioxidant assays, EF samples showed decreased IC_50_ values in DPPH and ABTS radical scavenging assays and increased FRAP values, indicating superior antioxidant capacity. In lipopolysaccharide (LPS)-stimulated RAW 264.7 macrophages, EF-treated leaf extracts effectively suppressed nitric oxide and intracellular ROS production without cytotoxicity and exhibited the highest GSH/GSSG ratio, suggesting improved cellular redox balance. In contrast, interleukin-6 (IL-6) secretion varied depending on tissue type and processing condition, indicating that antioxidant enhancement does not necessarily correspond to uniform cytokine regulation. Collectively, these findings demonstrate that enzymatic pretreatment combined with lactic acid fermentation serves as an effective strategy to enhance the antioxidant and cell-protective properties of radish by-products, supporting their potential use as value-added functional food ingredients.

## 1. Introduction

Radish (*Raphanus sativus* L.) is a root vegetable belonging to the family Brassicaceae and is widely cultivated in Asia, Europe, and North America, with the fleshy taproot primarily consumed as food [1,2]. Radish contains dietary fiber, vitamins, and minerals, as well as various phytochemical compounds such as polyphenols, flavonoids, and glucosinolates [3]. These compounds have been reported to be associated with antioxidant, anti-inflammatory, and antimicrobial activities [4]. In addition, the distribution and content of these functional components vary depending on radish tissues, with leaf tissues generally containing higher levels of phenolic compounds than root tissues [3].

During postharvest handling and distribution, the upper leaves are typically removed, and outer or damaged leaves are discarded to maintain product quality and storage stability [5]. As a result, a considerable amount of radish by-products is generated. Although these materials are edible, they are largely underutilized and have limited practical applications [6,7]. In particular, outer leaves removed during distribution and trimming residues contain substantial amounts of bioactive compounds, including polyphenols and flavonoids; however, their systematic utilization as functional food ingredients remains limited. Therefore, radish by-products can be considered an underutilized edible resource with potential for application as functional food materials.

Agricultural by-products contain various functional components, but their utilization is often limited due to technological constraints, leading to their disposal or use as low-value materials [6,8]. In recent years, increasing attention has been given to the development of value-added functional food materials through the evaluation of their chemical composition and biological activities [7,9]. Phenolic compounds in plant materials are often bound to cell wall polysaccharides, which restricts their efficient extraction using conventional solvent-based methods [7]. To address this limitation, enzymatic pretreatment has been applied to promote the release of bound compounds by degrading cell wall structures [10], which may enhance the efficiency of subsequent fermentation processes.

In addition, lactic acid fermentation has been reported to induce structural modification of plant-derived phenolic compounds through microbial enzymatic activity, potentially contributing to increased antioxidant activity [11,12]. In particular, *Lactiplantibacillus plantarum* is known to facilitate the biotransformation of phenolic compounds in plant-based substrates [12] and is widely used in fermentation processes for the development of functional food materials.

However, studies that systematically evaluate changes in functional components and biological activities following enzymatic pretreatment and lactic acid fermentation, either individually or in combination, in radish by-products remain limited. Moreover, comprehensive comparisons among different radish tissues, such as leaf blade, stem, and fleshy taproot, have rarely been reported. Unlike previous studies that have primarily focused on either single processing methods or limited plant tissues, the present study considers both tissue-specific differences and combined processing strategies in radish by-products [11,12]. Therefore, the objective of this study was to apply enzymatic pretreatment and *Lactiplantibacillus plantarum* fermentation, both individually and in combination, to radish by-products from different tissues (leaf blade, stem, and fleshy taproot). The physicochemical properties, phenolic and flavonoid contents, as well as antioxidant and anti-inflammatory activities were comparatively evaluated. In addition, this study aimed to examine tissue-specific responses and to explore the potential of radish by-products as functional food materials.

## 2. Materials and Methods

### 2.1. Materials

Radish (*Raphanus sativus* L.) samples used in this study consisted of radish by-products generated during harvesting and pre-distribution handling for commercial processing in Gyeongsangbuk-do, Republic of Korea. The collected radish by-products were separated into leaf blade (LB) and stem (ST) tissues, while fleshy taproots (RT) were separately prepared and used for comparative analysis. The radish (*Raphanus sativus* L.) samples used in this study were obtained from the ‘Cheongun’ cultivar cultivated in Andong, Gyeongsangbuk-do, Republic of Korea, in November 2024. For enzymatic pretreatment, cellulase derived from *Aspergillus niger* (≥0.3 U/mg solid) was used (Sigma-Aldrich, St. Louis, MO, USA). Fermentation was carried out using a food-grade lactic acid bacterium, *Lactiplantibacillus plantarum* KCTC 3108, obtained from the Korean Collection for Type Cultures (KCTC, Seoul, Republic of Korea). Ethanol and reagents for sodium acetate buffer preparation were purchased from Duksan Pure Chemicals (Ansan, Republic of Korea). Folin–Ciocalteu reagent, DPPH, ABTS, and FRAP reagents were obtained from Sigma-Aldrich (St. Louis, MO, USA) and were of analytical grade. MRS broth (Difco, Detroit, MI, USA) was used for lactic acid bacterial cultivation.

### 2.2. Processing and Extraction Conditions

Radish samples were classified according to tissue type and processing method and designated using abbreviated codes. Leaf blade, stem, and fleshy taproot samples were denoted as LB, ST, and RT, respectively. For each tissue, untreated control extracts were designated as C, enzyme-treated extracts as E, fermented extracts as F, and enzyme-treated and fermented extracts as EF. Accordingly, LB-C, LB-E, LB-F, and LB-EF represent the control, enzyme-treated, fermented, and enzyme-treated and fermented leaf blade extracts, respectively. The same nomenclature was applied to stem (ST-C–ST-EF) and fleshy taproot (RT-C–RT-EF) samples.

#### 2.2.1. Sample Pretreatment

Radish (*Raphanus sativus* L.) samples, including outer leaves and non-commercial leaf tissues removed prior to distribution, were collected and used in this study. The samples were washed under running tap water to remove surface impurities, followed by rinsing with distilled water. After pre-freezing at −40 °C, the samples were completely dried using a freeze dryer. The dried materials were ground into powder, sieved through a 60-mesh screen to obtain homogeneous powders, and stored in sealed containers at −20 °C until further analysis.

#### 2.2.2. Enzymatic Pretreatment

Freeze-dried radish and radish leaf powders were suspended in 50 mM sodium acetate buffer (pH 5.0) at a concentration of 5% (*w*/*v*). Cellulase derived from Aspergillus niger (≥0.3 U/mg solid) was added at 1% (*w*/*w*) relative to the substrate, corresponding to a final enzyme activity of ≥3 U/g substrate. The reaction mixture was incubated at 50 °C for 2 h with continuous agitation at 100 rpm. After enzymatic hydrolysis, the reaction was terminated by heating at 95 °C for 10 min to inactivate the enzyme. To ensure consistency across all groups and to exclude potential heat-induced effects, untreated control samples were subjected to the same heat treatment (95 °C for 10 min) without enzyme addition. The treated samples were then cooled to room temperature and subsequently subjected to extraction or fermentation.

#### 2.2.3. Fermentation Conditions

Fermentation of powdered radish tissues was conducted using *Lactiplantibacillus plantarum* KCTC 3108. The fermentation substrate was prepared by suspending powdered radish tissues in distilled water at a concentration of 5% (*w*/*v*), followed by sterilization at 121 °C for 15 min. The bacterial strain was activated by culturing in MRS broth for 18–24 h and inoculated into the sterilized substrate at 1% (*v*/*v*) to achieve an initial cell density of approximately 1 × 10^7^ CFU/mL. The inoculation level was selected to establish suitable fermentation conditions for radish by-products [9]. The fermentation time was established based on preliminary experiments conducted using radish leaf samples. Under these conditions, viable cell counts increased from approximately 1 × 10^7^ to 2 × 10^9^ CFU/mL after 24 h, accompanied by a decrease in pH from 6.3 to 6.0. Although organic acid production was not directly quantified in this study, these changes indicate that lactic acid fermentation proceeded effectively under the selected conditions. After fermentation, the samples were heated at 95 °C for 10 min to inactivate bacterial cells. For samples subjected to combined enzymatic pretreatment and fermentation (EF), cellulase treatment and enzyme inactivation were first performed, followed by pH adjustment to 6.0 using 0.1 N HCl or 0.1 N NaOH. The adjusted samples were then inoculated with *L. plantarum* and fermented under the same conditions.

#### 2.2.4. Preparation of Extracts for Analysis

**Figure 1:**
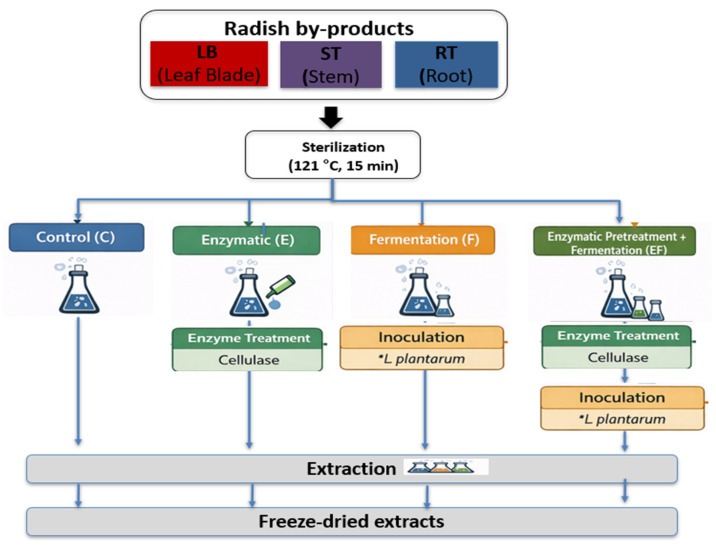
Experimental scheme for enzymatic pretreatment and fermentation of radish (*Raphanus sativus* L.) by-products. Samples were classified into LB, ST, and RT, sterilized (121 °C, 15 min), and subjected to control (C), enzymatic (E), fermentation (F), or combined (EF) treatments, followed by extraction and freeze-drying. * *L. plantarum*: *Lactiplantibacillus plantarum* used as the starter culture.

All samples subjected to enzymatic pretreatment and/or fermentation were extracted under identical conditions. Each treated sample was mixed with distilled water at a ratio of 1:10 (*w*/*v*) and extracted at 95 °C for 3 h under continuous agitation. Extraction was performed in triplicate for each sample, and the resulting extracts were combined and centrifuged at 8000× *g* for 20 min. The supernatants were filtered through No. 2 filter paper, concentrated using a rotary evaporator under reduced pressure, and subsequently freeze-dried. The dried extracts were stored at −20 °C until analysis (Figure 1).

### 2.3. Measurement of Quality Characteristics

#### 2.3.1. Yield, pH, and Reducing Sugar Content

The extraction yield was calculated based on the initial dried weight of plant materials, excluding added enzymes and microbial biomass. Extraction yield (%) was calculated as the ratio of dried extract weight to the initial dried sample weight. The pH of each extract was measured using a pH meter (CH-340; Mettler-Toledo Inc., Greinfensee, Switzerland) after preparing a 1% (*w*/*v*) aqueous solution and stirring at room temperature for 30 min. Reducing sugar content was determined using the 3,5-dinitrosalicylic acid (DNS) method [13]. The reaction mixture was heated in a boiling water bath for 5 min, cooled to room temperature, and the absorbance was measured at 540 nm using a UV–Vis spectrophotometer (UV-1800, Shimadzu Corp., Kyoto, Japan). Glucose was used as the standard, and the results were expressed as mg glucose equivalents per g extract.

#### 2.3.2. Total Polyphenol and Flavonoid Contents

Total polyphenol content was determined using the Folin–Ciocalteu method [14] with gallic acid as the standard. The results were expressed as mg gallic acid equivalents (GAE) per g extract. The calibration curve showed good linearity (y = 0.0019x + 0.0158, R^2^ = 0.9535). Total flavonoid content was measured using the aluminum chloride colorimetric method [15] with rutin as the standard and expressed as mg rutin equivalents (RE) per g extract. The calibration curve exhibited high linearity (y = 0.0061x − 0.0064, R^2^ = 0.9926).

#### 2.3.3. HPLC Analysis of Phenolic Compounds

Phenolic acid profiles were determined using an HPLC system (Prominence LC-20A series, Shimadzu Co., Kyoto, Japan) equipped with a quaternary pump, autosampler, column oven, and photodiode array detector. Separation was performed on a C18 reversed-phase column (250 × 4.6 mm, 5 μm; Shimadzu, Kyoto, Japan) at 30 °C. The mobile phase consisted of solvent A (2% acetic acid in water, *v*/*v*) and solvent B (methanol), delivered at a flow rate of 1.0 mL/min using a gradient elution program as follows: 0–10 min, 5–20% B; 10–30 min, 20–50% B; 30–40 min, 50–80% B; and 40–45 min, 80–5% B for re-equilibration. A 20 μL aliquot of each sample was injected, and detection wavelengths were set at 280 nm and 325 nm for the identification of phenolic compounds. Quantitative analysis was conducted based on external calibration curves constructed using authentic standards (gallic acid, chlorogenic acid, and ferulic acid; Sigma-Aldrich, St. Louis, MO, USA), and results were expressed as milligrams per gram of dried extract.

### 2.4. Antioxidant Activity Assays

#### 2.4.1. DPPH Radical Scavenging Activity

DPPH radical scavenging activity was evaluated according to the method of Blois [16]. The sample solution (40 μL) was mixed with 160 μL of 0.2 mM DPPH solution and incubated at 37 °C for 30 min in the dark. Absorbance was measured at 517 nm using a microplate reader (Infinite M200 Pro, Tecan, Männedorf, Switzerland). L-Ascorbic acid was used as the reference standard. IC_50_ values were calculated from three independent experiments by linear interpolation of the concentration–response data, and the resulting values were expressed as mean ± SD.

#### 2.4.2. ABTS Radical Scavenging Activity

ABTS radical scavenging activity was measured according to the method of Re et al. [17]. The ABTS^+^ solution was prepared by reacting 7 mM ABTS with 2.45 mM potassium persulfate and adjusted to an absorbance of 0.70 at 734 nm. The diluted ABTS^+^ solution (180 μL) was mixed with 40 μL of sample, and the absorbance was measured after 1 min. L-Ascorbic acid was used as the reference standard. IC_50_ values were calculated from three independent experiments by linear interpolation of the concentration–response data, and the resulting values were expressed as mean ± SD.

#### 2.4.3. FRAP Assay

Ferric reducing antioxidant power (FRAP) was determined according to the method of Benzie and Strain [18]. The FRAP reagent was prepared by mixing acetate buffer, 2,4,6-tripyridyl-s-triazine (TPTZ), and FeCl_3_·6H_2_O at a ratio of 10:1:1 (*v*/*v*/*v*). Each sample extract was adjusted to a fixed concentration of 400 μg/mL, reacted with the FRAP reagent at 37 °C for 5 min, and the absorbance was measured at 593 nm. The results were expressed as μmol FeSO_4_·7H_2_O equivalents per g extract, calculated from a FeSO_4_·7H_2_O standard calibration curve.

#### 2.4.4. Nitrite Scavenging Activity

Nitrite scavenging activity was evaluated according to the method of Gray and Dugan [19] with minor modifications. The reaction was conducted at pH 1.2 and 37 °C for 1 h, followed by the addition of Griess reagent. Absorbance was measured at 520 nm, and the activity was expressed as percentage inhibition.

#### 2.4.5. SOD-like Activity

SOD-like activity was determined by measuring the inhibition of pyrogallol autoxidation according to the method of Marklund and Marklund [20]. The change in absorbance was monitored at 420 nm, and the activity was expressed as percentage inhibition relative to the control.

#### 2.4.6. Catalase Activity

Catalase activity was measured based on the decomposition of hydrogen peroxide following the method of Aebi [21]. The decrease in absorbance at 240 nm was monitored, and the activity was expressed as relative activity (%) compared with the control. The concentration ranges for each assay were determined based on preliminary experiments to ensure that the measured responses fell within the appropriate linear range of each assay.

### 2.5. Anti-Inflammatory Activity

#### 2.5.1. Cell Culture and Sample Treatment

RAW 264.7 murine macrophages were obtained from the Korean Cell Line Bank (KCLB, Seoul, Republic of Korea) and cultured in Dulbecco’s modified Eagle’s medium (DMEM) supplemented with 10% fetal bovine serum (FBS) and 1% penicillin–streptomycin at 37 °C in a humidified incubator containing 5% CO_2_. Cells were seeded into 96-well plates and allowed to adhere overnight. Extracts were dissolved in dimethyl sulfoxide (DMSO), sterilized by filtration through a 0.22 μm membrane filter, and diluted with culture medium. The final concentration of DMSO in the culture medium was maintained below 0.1% (*v*/*v*). Cells were pretreated with the extracts for 1 h and subsequently stimulated with lipopolysaccharide (LPS, 1 μg/mL; *Escherichia coli* O111:B4, catalog no. L4391, Sigma-Aldrich, St. Louis, MO, USA) for 24 h.

#### 2.5.2. Cell Viability Assay

Cell viability was assessed using the MTT assay to determine non-cytotoxic concentrations of the extracts, as previously described by Mosmann [22]. RAW 264.7 macrophages were seeded in 96-well plates at a density of 1 × 10^4^ cells/well and treated with the extracts at concentrations ranging from 50 to 500 μg/mL for 24 h. After treatment, MTT solution (0.5 mg/mL) was added, and the plates were incubated for 4 h at 37 °C. The resulting formazan crystals were dissolved in DMSO, and absorbance was measured at 570 nm using a microplate reader (VersaMax, Molecular Devices, San Jose, CA, USA). Cell viability was expressed as a percentage relative to the untreated control. Based on preliminary cell viability screening, 300 μg/mL was selected as a non-cytotoxic concentration and applied in subsequent anti-inflammatory assays.

#### 2.5.3. Nitric Oxide (NO) Production Assay

Nitric oxide (NO) production was evaluated by measuring nitrite accumulation in the culture supernatant using the Griess reaction [23]. RAW 264.7 cells were pretreated with the extracts at 300 μg/mL for 1 h and then stimulated with LPS (1 μg/mL) for 24 h. Absorbance was measured at 540 nm, and nitrite concentration was calculated using a sodium nitrite standard curve. NO production was expressed as NO inhibition (%) relative to the LPS-treated control.

#### 2.5.4. Intracellular Reactive Oxygen Species (ROS) Measurement

Intracellular reactive oxygen species (ROS) levels were measured using the 2′,7′-dichlorodihydrofluorescein diacetate (DCFH-DA) assay [24]. After treatment with the extracts at 300 μg/mL, cells were stimulated with LPS (1 μg/mL) for 24 h prior to incubation with DCFH-DA (10 μM) for 30 min in the dark. Cells were then washed twice with phosphate-buffered saline (PBS), and fluorescence intensity was measured at excitation/emission wavelengths of 485/538 nm using a microplate reader (Spark 10M, Tecan, Männedorf, Switzerland). ROS levels were expressed as the percentage relative to the LPS-treated control.

#### 2.5.5. Cytokine Measurement

The concentrations of interleukin-6 (IL-6) in the culture supernatants were quantified using commercially available mouse ELISA kits (Thermo Fisher Scientific, Waltham, MA, USA), according to the manufacturer’s instructions. RAW 264.7 cells were treated with the extracts at 300 μg/mL and stimulated with lipopolysaccharide (LPS, 1 μg/mL) for 24 h. LPS-only and unstimulated cells were used as controls. Dexamethasone (10 μM) was included as a reference control. Absorbance was measured at 450 nm, and cytokine concentrations were first calculated from standard curves using a four-parameter logistic regression. The IL-6 levels were then normalized to the LPS-only group, which was set to 100%, and were expressed as a percentage of the LPS control.

#### 2.5.6. Intracellular GSH/GSSG Ratio

The intracellular GSH/GSSG ratio was determined using a GSH/GSSG Ratio Assay kit (ScienCell Research Laboratories, Carlsbad, CA, USA; Cat. No. 8558), according to the manufacturer’s instructions. RAW 264.7 cells were treated with the extracts at 300 μg/mL. Total glutathione and oxidized glutathione (GSSG) were measured spectrophotometrically at 412 nm. Reduced glutathione (GSH) was calculated as total glutathione minus twice the GSSG concentration (GSH = total glutathione − 2 × GSSG), and the GSH/GSSG ratio was expressed as GSH divided by GSSG.

### 2.6. Statistical Analysis

All experiments were performed independently in triplicate, and the results are presented as mean ± standard deviation (SD) (*n* = 3). Statistical comparisons were performed using one-way analysis of variance (ANOVA) followed by Duncan’s multiple range test using IBM SPSS Statistics (version 25.0; IBM Corp., Armonk, NY, USA). Samples were analyzed as independent groups according to tissue type and processing conditions. For phenolic acid composition, each compound was analyzed independently. IC_50_ values for DPPH and ABTS radical scavenging activities were calculated from three independent experiments and statistically compared in the same manner. Differences were considered statistically significant at *p* < 0.05.

## 3. Results

### 3.1. Physicochemical Properties and Bioactive Compound Contents

#### 3.1.1. pH, Reducing Sugar, Total Polyphenol, and Flavonoid Contents

The pH, reducing sugar content, total polyphenol content, and total flavonoid content of radish samples differed significantly depending on both the processing method and radish tissue type (Table 1, *p* < 0.05). An increase in reducing sugar content was generally observed in all radish tissues following treatments involving fermentation compared with the untreated controls, with the highest values observed in the enzyme-treated and fermented (EF) samples. In particular, EF treatment resulted in a significant increase in reducing sugar content in leaf blade (LB), stem (ST), and fleshy taproot (RT) samples, although the magnitude of the increase varied among tissues.

Total polyphenol and flavonoid contents also exhibited distinct differences according to the processing method. In all radish by-products, enzyme treatment and fermentation led to higher polyphenol and flavonoid contents than those of the control, while the EF treatment consistently showed the highest levels. When comparing different radish tissues, leaf blade samples generally contained higher amounts of total polyphenols and flavonoids than stem and fleshy taproot samples, and this tissue-dependent pattern was consistently observed across all treatment conditions.

#### 3.1.2. HPLC Analysis of Phenolic Compounds

The individual phenolic acid profiles of radish tissues (LB, ST, and RT) subjected to enzymatic pretreatment and fermentation were analyzed by HPLC, and the results are presented in Table 2. Chlorogenic acid was identified as the predominant phenolic acid in all samples, with its content showing significant differences depending on both radish tissue and processing method (*p* < 0.05). Overall, leaf blade (LB) samples exhibited higher chlorogenic acid contents than stem (ST) and fleshy taproot (RT) samples. In stem samples, enzymatic pretreatment followed by fermentation (EF) resulted in a significant increase in chlorogenic acid content. In contrast, fleshy taproot samples maintained relatively low levels of chlorogenic acid, and changes associated with processing treatments were limited. Caffeic acid, *p*-coumaric acid, ferulic acid, and sinapic acid also exhibited tissue- and treatment-dependent distribution patterns. Caffeic acid and ferulic acid were present at relatively higher levels in leaf blade samples, with increasing tendencies observed in some treatment groups. *p*-Coumaric acid was detected in all radish tissues; however, the magnitude of change according to processing method was relatively limited. Sinapic acid was detected only in leaf blade and stem samples and was not detected (N.D.) in fleshy taproot samples under any treatment condition. The sum of phenolic acids (Σ phenolic acids) was highest in leaf blade samples. In stem samples, Σ phenolic acids tended to increase following EF treatment, whereas in fleshy taproot samples, differences among treatment groups were limited.

### 3.2. Antioxidant Activity Assays

#### 3.2.1. DPPH Radical Scavenging Activity

**Figure 2:**
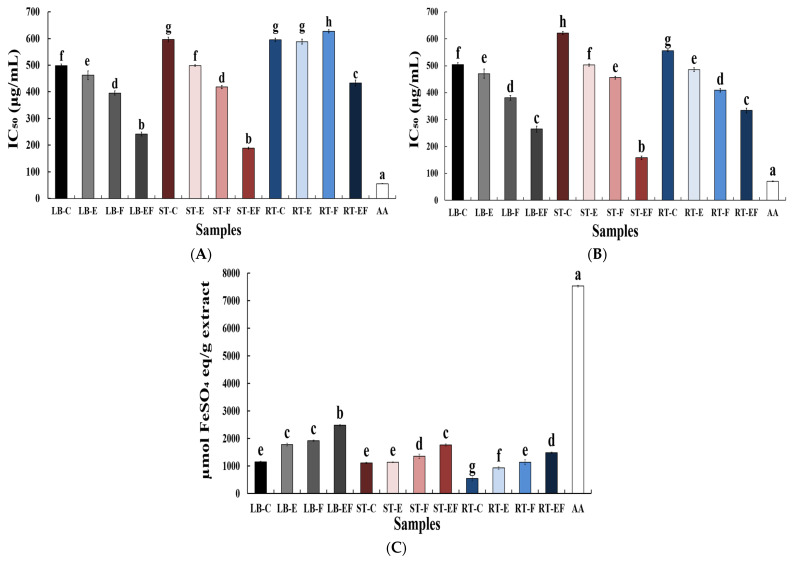
Antioxidant activities of radish samples as affected by enzymatic pretreatment and fermentation. (**A**) DPPH radical scavenging activity (IC_50_, µg/mL), (**B**) ABTS radical scavenging activity (IC_50_, µg/mL), and (**C**) ferric reducing antioxidant power (FRAP, µmol FeSO_4_ eq./g extract) at a fixed concentration. Different lowercase letters indicate significant differences among samples, as determined by one-way analysis of variance (ANOVA) followed by Duncan’s multiple range test (*p* < 0.05).

The DPPH radical scavenging activities of the samples were compared based on their IC_50_ values, and the results are presented in Figure 2A. In leaf blade (LB) samples, the untreated control (LB-C) exhibited an IC_50_ value of 498.34 μg/mL, whereas enzymatic pretreatment (LB-E) and fermentation (LB-F) reduced the IC_50_ values to 462.12 and 395.64 μg/mL, respectively. Notably, the combined enzymatic pretreatment and fermentation (LB-EF) resulted in the lowest IC_50_ value of 241.36 μg/mL, indicating a significant enhancement of DPPH radical scavenging activity. A similar trend was observed in stem (ST) samples. The ST-EF sample showed a markedly lower IC_50_ value (188.73 μg/mL) compared with the control (ST-C, 596.71 μg/mL). In fleshy taproot (RT) samples, RT-F exhibited the highest IC_50_ value (627.60 μg/mL), whereas the IC_50_ value decreased to 432.87 μg/mL in the RT-EF sample, demonstrating an improvement in antioxidant activity following the combined treatment.

#### 3.2.2. ABTS Radical Scavenging Activity

ABTS radical scavenging activity was evaluated based on the IC_50_ values of each sample, and the results are presented in Figure 2B. All samples exhibited a concentration-dependent increase in ABTS radical scavenging activity. In leaf blade (LB) samples, enzymatic pretreatment or fermentation reduced the IC_50_ values compared with the control, with the combined enzymatic pretreatment and fermentation (LB-EF) showing the most pronounced ABTS radical scavenging activity. A similar pattern was observed in stem (ST) samples, where both single treatments decreased IC_50_ values relative to the control, and the lowest IC_50_ value was observed in the enzyme-treated and fermented sample (ST-EF).

Notably, ST-EF exhibited the strongest ABTS radical scavenging activity among the radish samples tested. In fleshy taproot (RT) samples, IC_50_ values were generally higher than those of LB and ST samples. However, ABTS radical scavenging activity gradually improved following enzymatic pretreatment and fermentation, indicating a stepwise enhancement associated with the applied processing methods.

#### 3.2.3. Ferric Reducing Antioxidant Power (FRAP)

The FRAP results are shown in Figure 2C. In all radish by-products, ferric reducing power tended to increase following enzymatic pretreatment and fermentation. In leaf blade (LB) samples, FRAP values increased significantly after enzymatic pretreatment and fermentation compared with the control, with the highest reducing power observed in the enzyme-treated and fermented sample (LB-EF) (*p* < 0.05). In stem (ST) samples, no significant difference was observed between the control and enzyme-treated groups. In contrast, FRAP values increased significantly in the fermented (ST-F) and enzyme-treated and fermented (ST-EF) samples, with ST-EF exhibiting the highest reducing power among stem samples. Fleshy taproot (RT) samples showed overall lower FRAP values than LB and ST samples. Nevertheless, ferric reducing power gradually increased following enzymatic pretreatment and fermentation, and a significant enhancement was observed in the RT-EF sample.

#### 3.2.4. SOD and Catalase Activities

The SOD-like activity of all samples generally increased in a concentration-dependent manner over the range of 100–500 μg/mL (Table 3). In this study, statistical analyses were performed only to compare differences among treatment groups at the same concentration. In leaf blade (LB) samples, the enzyme-treated and fermented group (LB-EF) exhibited significantly higher SOD-like activity than the control and single-treatment groups at all tested concentrations (*p* < 0.05). Similar trends were observed in stem (ST) and fleshy taproot (RT) samples, where the EF-treated groups consistently showed the highest SOD-like activity at the same concentration, indicating a uniform treatment effect across different radish tissues.

Catalase (CAT) activity also showed an increasing tendency with increasing concentration (Table 3), with relatively higher activities observed in the enzyme-treated and fermented groups. In particular, the EF-treated LB samples maintained higher CAT activity across the entire concentration range. In stem (ST) samples, the EF-treated group exhibited significantly higher CAT activity than the control and single-treatment groups at higher concentrations (*p* < 0.05).

To further investigate the relationship between bioactive compounds and antioxidant activity, Pearson correlation analysis was conducted (Table S1). Total polyphenol and flavonoid contents showed strong positive correlations with FRAP values (r = 0.952 and 0.939, respectively) and strong negative correlations with DPPH IC_50_ values (r = −0.916 and −0.907, respectively). In addition, DPPH and ABTS IC_50_ values were highly correlated (r = 0.964), indicating similar radical scavenging behavior. These results suggest that phenolic compounds play a key role in the antioxidant activity of radish by-products.

### 3.3. Anti-Inflammatory Effects in RAW 264.7 Macrophages

#### 3.3.1. Cell Viability

**Figure 3:**
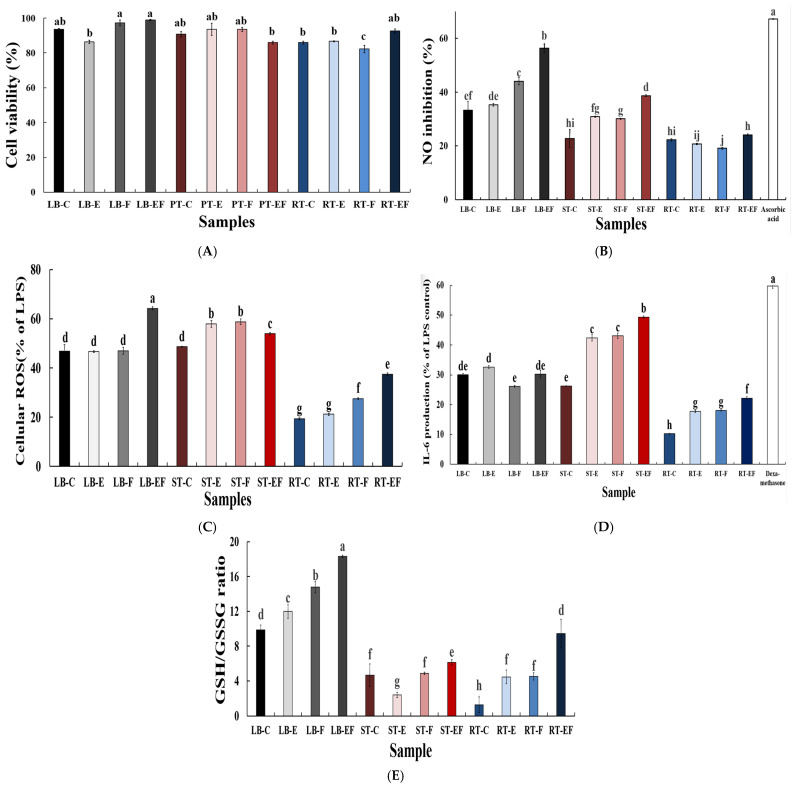
Anti-inflammatory and redox-related effects of radish extracts in LPS-stimulated RAW 264.7 macrophages. (**A**) Cell viability (%), (**B**) nitric oxide (NO) inhibition (%), (**C**) intracellular reactive oxygen species (ROS) levels (% of LPS-treated control), (**D**) interleukin-6 (IL-6) secretion (% of LPS-treated control), and (**E**) intracellular GSH/GSSG ratio in RAW 264.7 macrophages treated with radish extracts at 300 μg/mL. Data are presented as mean ± SD (*n* = 3). Different lowercase letters indicate significant differences among samples, as determined by one-way analysis of variance (ANOVA) followed by Duncan’s multiple range test (*p* < 0.05).

To evaluate the cytotoxicity of the extracts, cell viability was first assessed in RAW 264.7 macrophages at concentrations ranging from 100 to 500 μg/mL. As a result, all samples maintained cell viability above 80% up to 300 μg/mL, indicating no significant cytotoxic effects within this concentration range. Based on these findings, 300 μg/mL was selected as an appropriate concentration for subsequent anti-inflammatory assays. The cell viability results at this concentration are presented in Figure 3A.

#### 3.3.2. NO Inhibition (%) in LPS-Stimulated RAW 264.7 Macrophages

The inhibitory effects of the samples on nitric oxide (NO) production in LPS-stimulated RAW 264.7 macrophages are shown in Figure 3B. All treated groups exhibited inhibitory effects on NO production, with significant differences observed among samples (*p* < 0.05). Among the leaf blade (LB) samples, the enzyme-treated and fermented group (LB-EF) showed the highest NO inhibition (57.35%), which was significantly higher than that of the control (LB-C, 33.24%) and the single-treatment groups (LB-E, 35.30%; LB-F, 44.08%). In stem (ST) samples, the ST-EF group exhibited an increased NO inhibitory effect (38.70%) compared with the control (ST-C, 22.77%). In contrast, fleshy taproot (RT) samples showed relatively low NO inhibition, ranging from 19.14% to 24.14%, regardless of the processing method.

#### 3.3.3. Cellular ROS Inhibition

The inhibition of intracellular reactive oxygen species (ROS) generation in LPS-stimulated RAW 264.7 macrophages is shown in Figure 3C and differed significantly depending on the sample treatment (*p* < 0.05). In leaf blade (LB) samples, the control (LB-C) and the single-treatment groups (LB-E and LB-F) exhibited comparable levels of ROS inhibition, with no significant differences among them. In contrast, the enzyme-treated and fermented group (LB-EF) showed a significantly higher ROS inhibitory effect than the non-fermented LB samples. In stem (ST) samples, the enzyme-treated (ST-E) and fermented (ST-F) groups exhibited significantly higher ROS inhibition than the control (ST-C). The ST-EF group showed an intermediate level of ROS inhibition, higher than that of ST-C but lower than those of ST-E and ST-F. Fleshy taproot (RT) samples generally exhibited lower ROS inhibitory activity than LB and ST samples. RT-C and RT-E showed the lowest inhibition levels, whereas RT-F and RT-EF exhibited significantly increased ROS inhibition compared with the non-fermented RT samples.

#### 3.3.4. Cytokine Production

The levels of interleukin-6 (IL-6) secretion in LPS-stimulated RAW 264.7 macrophages treated with radish tissue extracts are presented in Figure 3D. In leaf blade (LB) samples, the fermented group (LB-F) showed lower IL-6 secretion than the control (LB-C), whereas the enzyme-treated (LB-E) and enzyme-treated and fermented (LB-EF) groups maintained levels comparable to those of the control. In stem (ST) samples, IL-6 secretion increased markedly depending on the processing method, with the highest level observed in the enzyme-treated and fermented group (ST-EF). Fleshy taproot (RT) samples exhibited overall lower IL-6 secretion levels than LB and ST samples across all treatment groups; however, a gradual increase in IL-6 secretion was observed following enzymatic pretreatment and fermentation.

#### 3.3.5. Intracellular GSH/GSSG Ratio in LPS-Stimulated RAW 264.7 Cells

The intracellular GSH/GSSG ratios of cells treated with the extracts at 300 μg/mL are shown in Figure 3E. Significant differences were observed depending on radish tissue type and processing method (*p* < 0.05). In leaf blade (LB) samples, the enzyme-treated (LB-E) and fermented (LB-F) groups showed increased GSH/GSSG ratios compared with the control (LB-C), with the highest value observed in the enzyme-treated and fermented group (LB-EF). In LB samples, the GSH/GSSG ratio generally increased with successive processing steps. In stem (ST) samples, the GSH/GSSG ratios ranged from 2.16 to 4.89 and were generally lower than those observed in LB samples. ST-C and ST-F exhibited comparable values, whereas relatively lower GSH/GSSG ratios were maintained in ST-E and ST-EF. In fleshy taproot (RT) samples, the control group (RT-C) showed the lowest GSH/GSSG ratio, while a significant increase was observed in the enzyme-treated and fermented group (RT-EF). These results indicate that the intracellular GSH/GSSG response varied depending on radish tissue, even under identical processing conditions.

## 4. Discussion

In this study, radish by-products were classified according to tissue type, and the effects of enzymatic pretreatment, lactic acid fermentation, and their combined application (EF) on physicochemical properties, antioxidant activities, and cell-based anti-inflammatory and redox-related indicators were systematically compared. Overall, the EF-treated samples exhibited more consistent functional improvements than the single-treatment groups, indicating that the combined application of enzymatic pretreatment and fermentation resulted in relatively enhanced biofunctional properties. Taken together, these findings suggest that enzymatic pretreatment plays a critical role, rather than merely an auxiliary step, in facilitating fermentation-driven functional enhancement of radish by-products [9,10]. The decrease in pH observed in the EF-treated samples, together with the increased reducing sugar, polyphenol, and flavonoid contents, may be attributed to cellulase-mediated cell wall hydrolysis. This process likely enhances substrate accessibility by disrupting plant cell wall structures, thereby facilitating microbial enzyme activity and accelerating metabolic conversion during subsequent fermentation. Such a role of enzymatic pretreatment is consistent with previous studies reporting that enzymatic disruption of plant cell walls enhances substrate accessibility and metabolic efficiency throughout fermentation processes [10].

All fermentation samples were subjected to identical sterilization conditions (121 °C, 15 min), suggesting that the observed differences are primarily associated with enzymatic and microbial treatments. Enzymatic pretreatment has been reported to promote the release of phenolic compounds bound to plant cell wall matrices, while lactic acid fermentation may induce structural modification and partial depolymerization of phenolic compounds through microbial enzyme activities [8,11,12]. These changes may contribute to the improved extractability of phenolic compounds and the enhanced antioxidant activity observed in this study. In the present study, leaf blade (LB) by-products exhibited a decrease in chlorogenic acid content accompanied by increases in caffeic acid and ferulic acid following EF treatment, suggesting a possible transformation of caffeoylquinic acid-related compounds during fermentation. Similar phenolic acid conversion patterns have been reported in plant leaf tissues subjected to enzymatic and fermentation treatments [25,26]. In contrast, such compositional changes were less pronounced in fleshy taproot (RT) tissues, indicating that tissue-specific differences in cell wall structure and storage composition influence enzymatic and fermentation responsiveness. In antioxidant activity assays, EF-treated samples consistently exhibited the strongest activities across all tissue types, as determined by DPPH, ABTS, and FRAP assays. These results reflect not only quantitative increases in antioxidant components but also functional improvements associated with structural modifications induced by the combined enzymatic and fermentation processes. Given that DPPH, ABTS, and FRAP assays are based on different reaction mechanisms, the combined evaluation using multiple assays enhances the reliability of antioxidant activity assessment [27]. Photosynthetic tissues such as leaves and stems are known to accumulate higher levels of antioxidant defense systems and phenolic compounds due to continuous exposure to photo-oxidative stress [3,28]. In the present study, Pearson correlation analysis demonstrated that total polyphenol and flavonoid contents were strongly associated with antioxidant activity, as reflected by their relationships with FRAP values and DPPH IC_50_.

In RAW 264.7 macrophage experiments, all samples maintained cell viability within a non-cytotoxic range up to 300 μg/mL. Under these conditions, the observed inhibition of NO production and intracellular ROS generation can be attributed to the intrinsic bioactivities of the samples rather than to cytotoxic effects. Notably, the enhanced ROS inhibition and improved GSH/GSSG ratios observed in EF-treated samples suggest that the combined enzymatic and fermentation process may be associated with modulation of intracellular redox status under inflammatory stimulation [27,28]. This effect is likely associated with the enhanced release and improved cellular availability of phenolic compounds during enzymatic treatment and fermentation [29,30,31], which may contribute to the modulation of redox-sensitive signaling pathways involved in inflammatory and oxidative stress responses [32,33].

In contrast, IL-6 secretion did not consistently decrease across all samples and exhibited tissue- and treatment-dependent patterns. In leaf tissues, fermentation treatment tended to reduce IL-6 secretion relative to the control, indicating a potential modulatory effect under specific processing conditions. These findings show a similar tendency to previous studies reporting that plant-derived phenolic compounds can influence cytokine production in macrophages [33,34]. However, stem and taproot tissues did not show a clear reduction in IL-6 secretion under identical treatment conditions, suggesting that enhanced antioxidant activity does not necessarily correspond to uniform suppression of inflammatory cytokine production. This discrepancy may be attributed to differences in phenolic composition rather than total phenolic content. In particular, leaf tissues are known to contain higher levels of chlorogenic acid, which has been reported to modulate inflammatory signaling pathways such as NF-κB activation and cytokine production. Therefore, the tissue-specific IL-6 responses observed in this study may be influenced by qualitative differences in phenolic composition rather than overall antioxidant capacity. In addition, these findings suggest that specific phenolic constituents present in each tissue may selectively modulate inflammatory signaling pathways, thereby contributing to differential regulation of IL-6 production.

Furthermore, the GSH/GSSG ratio analysis demonstrated that intracellular redox homeostasis was differentially regulated depending on tissue type and processing method. The GSH/GSSG ratio is a widely recognized indicator of cellular redox status, reflecting the balance between reduced and oxidized glutathione, and is closely associated with cellular defense mechanisms and inflammatory responses [32]. In the present study, EF-treated leaf samples exhibited the highest GSH/GSSG ratios, suggesting that the combined enzymatic and fermentation process may enhance the cellular availability and functional efficacy of antioxidant components. Fermentation has been reported to induce deglycosylation and structural modification of phenolic compounds, thereby improving cellular accessibility [33,34]. These changes may further enhance intracellular antioxidant efficiency and redox regulation capacity and may be partially associated with the differences in redox responses observed in this study.

Overall, these findings highlight that the integration of enzymatic pretreatment and lactic acid fermentation represents an effective strategy for enhancing the functional value of radish by-products through improved phenolic accessibility, biotransformation, and redox-regulating capacity. In particular, the differential regulation of IL-6 suggests that anti-inflammatory activity may be more strongly influenced by qualitative differences in phenolic composition than by total antioxidant capacity. These findings highlight the importance of enzymatic pretreatment as a key preparatory step in fermentation-based processing strategies for the functional utilization of radish by-products. However, since molecular-level mechanisms were not directly investigated in this study, further research focusing on antioxidant enzyme systems and related signaling pathways is warranted to clarify the underlying mechanisms.

## 5. Conclusions

In this study, the antioxidant and anti-inflammatory functional properties of radish (*Raphanus sativus* L.) by-products were comparatively evaluated according to tissue type following cellulase pretreatment and *Lactiplantibacillus plantarum* fermentation, applied individually or in combination. The combined enzymatic pretreatment and fermentation (EF) consistently resulted in the most pronounced enhancement of antioxidant activity and bioactive compound content across all radish by-products, with the effects being most evident in leaf by-products. EF treatment significantly increased total polyphenol and flavonoid contents and led to an overall improvement in antioxidant capacity, as demonstrated by reduced IC_50_ values in DPPH and ABTS radical scavenging assays and increased FRAP values. In addition, SOD- and catalase-related activities were more stably enhanced in EF-treated samples, indicating that the combined processing approach contributed to improvements across multiple antioxidant defense indicators. In cell-based assays using RAW 264.7 macrophages, EF-treated leaf samples effectively suppressed LPS-induced NO production and intracellular ROS generation without inducing cytotoxicity. In contrast, IL-6 secretion responses varied depending on tissue type and processing condition, suggesting that enhanced antioxidant activity does not uniformly translate into suppression of inflammatory cytokine production. These findings indicate that anti-inflammatory evaluation should be interpreted in consideration of both tissue-specific characteristics and processing methods rather than relying on a single biomarker. Unlike previous studies that have mainly focused on individual treatments or single plant tissues, this study demonstrates that the combined application of enzymatic pretreatment and fermentation across different radish tissues provides a more integrated approach to enhancing functional properties. Overall, this study experimentally demonstrates that radish by-products, which are typically discarded during pre-distribution handling and regarded as low-value materials, can be converted into functional food ingredients through enzymatic pretreatment combined with lactic acid fermentation. The results provide fundamental evidence supporting the sustainable, value-added utilization of agricultural by-products and offer practical insights for the development of processing strategies targeting functional food applications.

## Figures and Tables

**Table 1:** Physicochemical properties and bioactive compound contents of radish samples as affected by enzymatic pretreatment and fermentation.

Sample	Yield (%)	pH	Reducing Sugar (mg Glucose eq./g)	Polyphenols (mg GAE/g)	Flavonoids (mg RE/g)
LB-C	10.58 ± 0.69 ^e^	6.33 ± 0.07 ^d^	74.21 ± 0.31 ^b^	187.25 ± 2.83 ^c^	75.47 ± 0.73 ^c^
LB-E	11.64 ± 1.82 ^e^	6.19 ± 0.04 ^e^	69.84 ± 0.32 ^c^	194.61 ± 2.39 ^b^	77.64 ± 0.73 ^b^
LB-F	11.77 ± 1.06 ^e^	5.97 ± 0.06 ^f^	69.42 ± 0.50 ^c^	203.63 ± 3.86 ^a^	79.77 ± 0.64 ^b^
LB-EF	16.82 ± 1.68 ^b^	5.16 ± 0.17 ^g^	92.89 ± 1.23 ^a^	212.84 ± 2.85 ^a^	81.78 ± 0.65 ^a^
ST-C	12.88 ± 1.75 ^d^	7.28 ± 0.01 ^a^	55.64 ± 0.89 ^e^	126.86 ± 2.83 ^f^	61.64 ± 0.73 ^e^
ST-E	13.56 ± 1.63 ^cd^	7.29 ± 0.00 ^a^	56.45 ± 0.32 ^e^	134.41 ± 2.86 ^e^	63.64 ± 0.73 ^d^
ST-F	12.63 ± 0.21 ^d^	7.04 ± 0.01 ^b^	58.02 ± 1.88 ^d^	141.86 ± 2.68 ^d^	65.66 ± 0.73 ^d^
ST-EF	15.56 ± 0.08 ^b^	6.87 ± 0.03 ^c^	72.94 ± 1.98 ^b^	149.37 ± 2.27 ^c^	73.46 ± 0.73 ^c^
RT-C	11.66 ± 1.16 ^e^	7.02 ± 0.02 ^b^	31.58 ± 0.20 ^h^	31.14 ± 2.31 ^h^	33.34 ± 1.00 ^h^
RT-E	13.21 ± 2.32 ^cd^	7.18 ± 0.06 ^b^	33.03 ± 0.27 ^g^	78.69 ± 2.33 ^g^	36.12 ± 0.71 ^g^
RT-F	13.99 ± 2.49 ^c^	6.84 ± 0.01 ^c^	55.15 ± 0.30 ^e^	85.84 ± 2.33 ^f^	46.62 ± 0.64 ^f^
RT-EF	17.85 ± 1.82 ^a^	6.36 ± 0.04 ^d^	99.18 ± 0.36 ^a^	120.88 ± 1.91 ^e^	51.86 ± 0.72 ^e^

Values are expressed as mean ± SD (*n* = 3). LB, leaf blade; ST, stem; RT, fleshy taproot; C, control; E, enzymatic pretreatment; F, fermentation; EF, enzymatic pretreatment followed by fermentation. Different lowercase letters within the same column indicate significant differences among all samples, as determined by one-way ANOVA followed by Duncan’s multiple range test (*p* < 0.05).

**Table 2:** Phenolic acid composition of radish samples as affected by tissue type and treatment method.

Sample	Chlorogenic Acid	Caffeic Acid	*p*-Coumaric Acid	Ferulic Acid	Sinapic Acid	Σ Phenolic Acids
LB-C	13.34 ± 0.41 ^a^	2.46 ± 0.35 ^d^	2.31 ± 0.43 ^b^	1.83 ± 0.20 ^b^	0.81 ± 0.12 ^a^	20.75 ± 0.21 ^a^
LB-E	11.41 ± 0.48 ^b^	3.48 ± 0.09 ^c^	3.06 ± 0.41 ^a^	1.17 ± 0.13 ^c^	0.63 ± 0.39 ^ab^	19.74 ± 0.91 ^b^
LB-F	12.37 ± 0.20 ^ab^	3.54 ± 0.44 ^c^	2.23 ± 0.19 ^b^	1.62 ± 0.21 ^b^	0.54 ± 0.08 ^b^	20.29 ± 0.30 ^ab^
LB-EF	9.87 ± 0.26 ^c^	5.43 ± 0.16 ^a^	2.93 ± 0.32 ^a^	2.32 ± 0.31 ^a^	0.60 ± 0.02 ^ab^	21.15 ± 0.79 ^a^
ST-C	5.50 ± 0.26 ^e^	1.61 ± 0.31 ^e^	0.73 ± 0.07 ^c^	0.66 ± 0.03 ^d^	0.28 ± 0.05 ^c^	8.78 ± 0.39 ^c^
ST-E	5.23 ± 0.14 ^e^	1.72 ± 0.21 ^e^	0.95 ± 0.02 ^c^	0.46 ± 0.02 ^d^	0.86 ± 0.09 ^a^	9.22 ± 0.38 ^c^
ST-F	6.51 ± 0.44 ^d^	1.56 ± 0.13 ^e^	0.83 ± 0.09 ^c^	0.86 ± 0.01 ^c^	0.55 ± 0.16 ^b^	10.30 ± 0.14 ^c^
ST-EF	8.48 ± 0.28 ^c^	1.83 ± 0.41 ^e^	1.38 ± 0.14 ^c^	0.84 ± 0.04 ^c^	0.89 ± 0.06 ^a^	13.42 ± 0.67 ^b^
RT-C	4.61 ± 3.34 ^e^	1.59 ± 0.51 ^e^	1.33 ± 0.24 ^c^	0.69 ± 0.03 ^cd^	N.D.	8.22 ± 3.46 ^c^
RT-E	3.54 ± 0.28 ^e^	1.31 ± 0.16 ^e^	1.67 ± 0.10 ^b^	0.67 ± 0.23 ^cd^	N.D.	7.20 ± 0.29 ^c^
RT-F	3.31 ± 0.27 ^e^	1.58 ± 0.33 ^e^	1.77 ± 0.10 ^b^	0.35 ± 0.14 ^d^	N.D.	7.01 ± 0.54 ^c^
RT-EF	3.47 ± 0.39 ^e^	1.29 ± 0.14 ^e^	1.44 ± 0.24 ^c^	0.90 ± 0.07 ^c^	N.D.	7.10 ± 0.22 ^c^

Values are expressed as mean ± standard deviation (SD) (*n* = 3). LB, leaf blade; ST, stem; RT, fleshy taproot; C, control; E, enzymatic pretreatment; F, fermentation; EF, enzymatic pretreatment followed by fermentation. Different lowercase letters within the same column indicate significant differences among all samples, as determined by one-way ANOVA followed by Duncan’s multiple range test (*p* < 0.05). N.D., not detected under the present analytical conditions.

**Table 3:** SOD and catalase activities of radish extracts as affected by enzymatic pretreatment and fermentation.

	SOD-like Activity (% Inhibition)			Catalase Activity (Relative Activity, %)		
Sample	100 μg/mL	300 μg/mL	500 μg/mL	100 μg/mL	200 μg/mL	400 μg/mL
LB-C	29.32 ± 0.81 ^g^	51.33 ± 0.81 ^e^	63.58 ± 1.12 ^d^	44.80 ± 0.30 ^c^	60.19 ± 0.14 ^b^	63.07 ± 1.01 ^ef^
LB-E	32.20 ± 0.52 ^f^	56.37 ± 0.15 ^d^	65.73 ± 0.18 ^c^	32.67 ± 0.12 ^d^	50.19 ± 0.24 ^c^	47.01 ± 0.21 ^g^
LB-F	25.36 ± 0.21 ^h^	29.72 ± 1.24 ^f^	48.48 ± 0.18 ^e^	46.85 ± 0.16 ^b^	63.42 ± 0.09 ^b^	70.51 ± 0.15 ^cd^
LB-EF	48.84 ± 0.11 ^c^	76.90 ± 0.11 ^b^	81.79 ± 0.49 ^a^	63.25 ± 1.08 ^b^	73.30 ± 0.81 ^a^	82.47 ± 0.14 ^b^
ST-C	28.38 ± 2.92 ^g^	43.86 ± 1.38 ^e^	64.54 ± 2.30 ^cd^	21.19 ± 0.63 ^f^	35.76 ± 1.44 ^e^	46.50 ± 3.24 ^g^
ST-E	39.30 ± 1.32 ^d^	50.69 ± 0.60 ^e^	55.69 ± 0.63 ^e^	24.08 ± 0.46 ^ef^	39.80 ± 1.33 ^d^	60.97 ± 0.74 ^ef^
ST-F	37.19 ± 0.47 ^d^	50.69 ± 0.60 ^e^	62.94 ± 0.42 ^d^	26.77 ± 1.45 ^e^	43.60 ± 3.29 ^d^	51.70 ± 0.06 ^g^
ST-EF	57.01 ± 0.42 ^b^	52.63 ± 0.63 ^e^	75.21 ± 0.71 ^b^	23.62 ± 0.95 ^ef^	43.20 ± 1.06 ^d^	76.55 ± 2.93 ^bc^
RT-C	40.84 ± 2.75 ^d^	54.32 ± 2.88 ^e^	50.35 ± 2.16 ^e^	20.37 ± 0.19 ^f^	24.38 ± 0.26 ^f^	54.87 ± 0.77 ^g^
RT-E	45.72 ± 0.98 ^c^	56.00 ± 1.43 ^d^	76.73 ± 1.80 ^b^	20.81 ± 0.08 ^f^	43.11 ± 2.61 ^d^	59.30 ± 2.11 ^f^
RT-F	37.51 ± 0.79 ^d^	46.19 ± 0.51 ^e^	52.99 ± 1.01 ^e^	38.99 ± 0.94 ^c^	58.30 ± 2.45 ^b^	66.63 ± 3.50 ^de^
RT-EF	45.84 ± 0.80 ^c^	54.26 ± 0.11 ^e^	83.51 ± 2.97 ^a^	44.16 ± 2.37 ^c^	52.44 ± 0.95 ^c^	65.62 ± 0.15 ^d^
Ascorbic acid	79.56 ± 0.23 ^a^	88.66 ± 0.32 ^a^	97.05 ± 0.79 ^a^	73.76 ± 0.16 ^a^	80.98 ± 0.55 ^a^	89.71 ± 3.08 ^a^

Values are expressed as mean ± SD (*n* = 3). Different lowercase letters within the same column indicate significant differences among all samples, as determined by one-way ANOVA followed by Duncan’s multiple range test (*p* < 0.05).

## Data Availability

The original contributions presented in this study are included in the article. Further inquiries can be directed to the corresponding author.

## Supplementary Materials

The following supporting information can be downloaded at https://www.mdpi.com/article/10.3390/foods15071150/s1, Table S1: Pearson correlation coefficients among bioactive compounds and antioxidant activities of radish by-products. Values represent correlation coefficients (r).

## Abbreviations

The following abbreviations are used in this manuscript:

ABTS	2,2′-azino-bis(3-ethylbenzothiazoline-6-sulfonic acid)
DMEM	Dulbecco’s modified Eagle’s medium
DPPH	2,2-diphenyl-1-picrylhydrazyl
ELISA	Enzyme-linked immunosorbent assay
FBS	Fetal bovine serum
FRAP	Ferric reducing antioxidant power
GAE	Gallic acid equivalents
HPLC	High-performance liquid chromatography
IL-6	Interleukin-6
LPS	Lipopolysaccharide
MTT	3-(4,5-dimethylthiazol-2-yl)-2,5-diphenyltetrazolium bromide
NO	Nitric oxide
PBS	Phosphate-buffered saline
RE	Rutin equivalents
ROS	Reactive oxygen species
SOD	Superoxide dismutase
