# Supplementary material for: Enhancement of Antioxidant and Anti-Inflammatory Activities of Radish (Raphanus sativus L.) By-Products Through Enzymatic Pretreatment and Lactic Acid Fermentation
Source: Foods. 2026 Mar 27;15(7):1150. doi: 10.3390/foods15071150 (PMC13072732; doi:10.3390/foods15071150)
Supplement: Supplementary file 1 [file foods-15-01150-s001.zip › foods-4207588-supplementary.pdf]

Table S1. Pearson correlation coefficients among bioactive compounds and antioxidant activities of radish by-products. Values represent correlation coefficients (r).

Parameter	Flavonoid	Polyphenol	DPPH IC_{50}	ABTS IC_{50}	FRAP	SOD	CAT
Flavonoid	1						
Polyphenol	0.981**	1					
DPPH IC_{50}	-0.907**	-0.916**	1				
ABTS IC_{50}	-0.876**	-0.895**	0.964**	1			
FRAP	0.939**	0.952**	-0.907**	-0.887**	1		
SOD	0.742**	0.754**	-0.772**	-0.735**	0.828**	1	
CAT	0.681**	0.701**	-0.650*	-0.620*	0.771**	0.720**	1

Values represent correlation coefficients (r). **$p < 0.01$, *$p < 0.05$.
